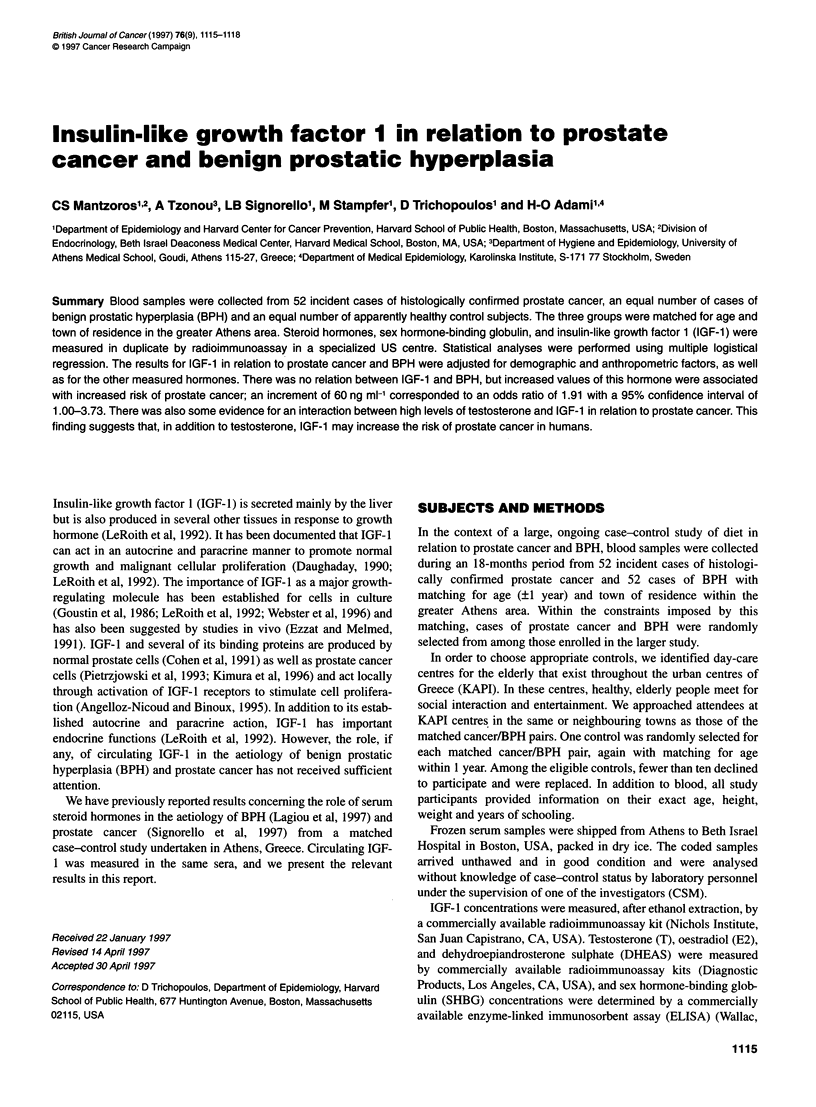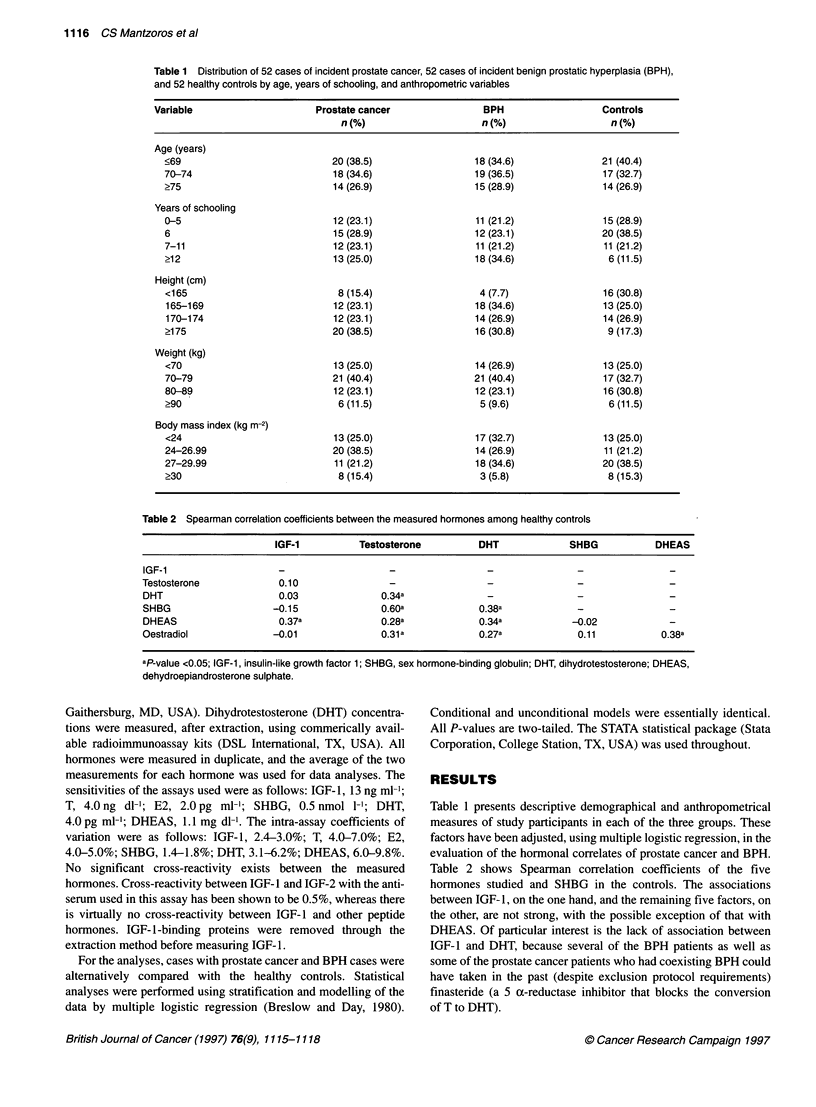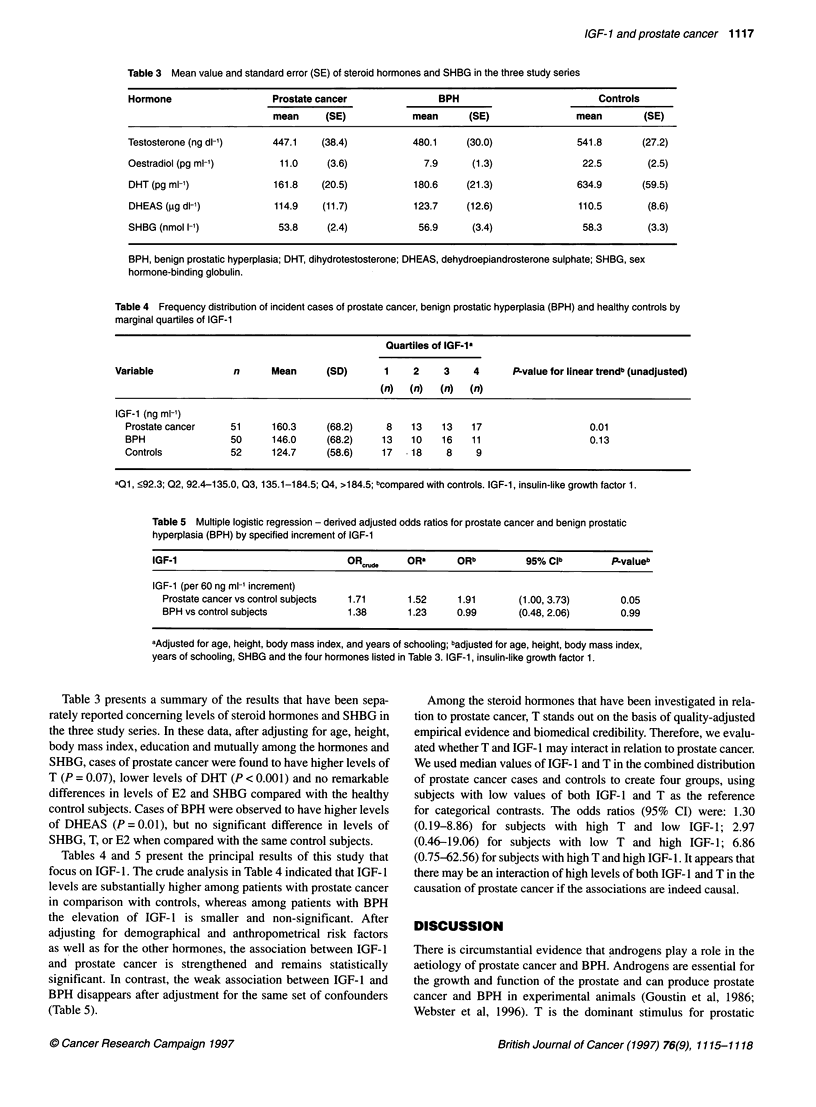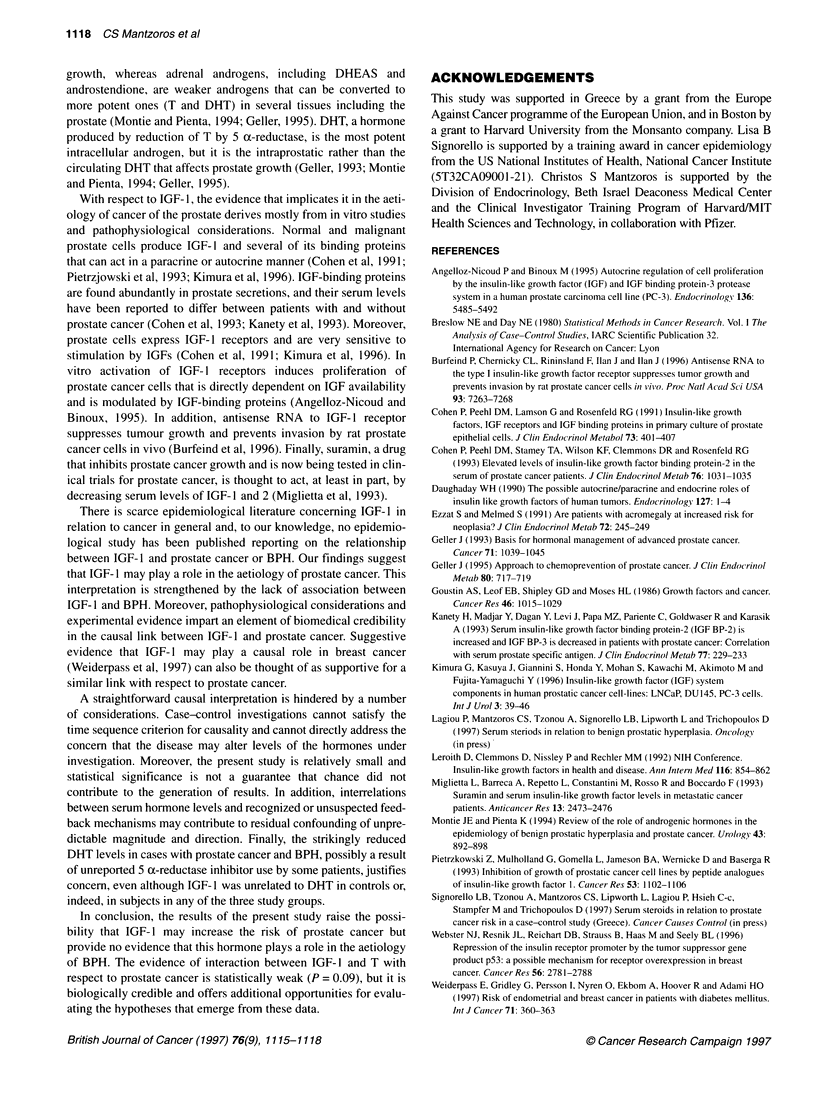# Insulin-like growth factor 1 in relation to prostate cancer and benign prostatic hyperplasia.

**DOI:** 10.1038/bjc.1997.520

**Published:** 1997

**Authors:** C. S. Mantzoros, A. Tzonou, L. B. Signorello, M. Stampfer, D. Trichopoulos, H. O. Adami

**Affiliations:** Department of Epidemiology and Harvard Center for Cancer Prevention, Harvard School of Public Health, Boston, Massachusetts 02115, USA.

## Abstract

Blood samples were collected from 52 incident cases of histologically confirmed prostate cancer, an equal number of cases of benign prostatic hyperplasia (BPH) and an equal number of apparently healthy control subjects. The three groups were matched for age and town of residence in the greater Athens area. Steroid hormones, sex hormone-binding globulin, and insulin-like growth factor 1 (IGF-1) were measured in duplicate by radioimmunoassay in a specialized US centre. Statistical analyses were performed using multiple logistical regression. The results for IGF-1 in relation to prostate cancer and BPH were adjusted for demographic and anthropometric factors, as well as for the other measured hormones. There was no relation between IGF-1 and BPH, but increased values of this hormone were associated with increased risk of prostate cancer; an increment of 60 ng ml(-1) corresponded to an odds ratio of 1.91 with a 95% confidence interval of 1.00-3.73. There was also some evidence for an interaction between high levels of testosterone and IGF-1 in relation to prostate cancer. This finding suggests that, in addition to testosterone, IGF-1 may increase the risk of prostate cancer in humans.


British Journal of Cancer (1997) 76(9), 1115–1118
© 1997 Cancer Research Campaign

### Insulin-like growth factor 1 in relation to prostate cancer and benign prostatic hyperplasia

**CS Mantzoros[1,2], A Tzonou[3], LB Signorello[1], M Stampfer[1], D Trichopoulos[1] and H-O Adami[1,4]**

[1]Department of Epidemiology and Harvard Center for Cancer Prevention, Harvard School of Public Health, Boston, Massachusetts, USA; [2]Division of Endocrinology, Beth Israel Deaconess Medical Center, Harvard Medical School, Boston, MA, USA; [3]Department of Hygiene and Epidemiology, University of Athens Medical School, Goudi, Athens 115-27, Greece; [4]Department of Medical Epidemiology, Karolinska Institute, S-171 77 Stockholm, Sweden

**Summary** Blood samples were collected from 52 incident cases of histologically confirmed prostate cancer, an equal number of cases of benign prostatic hyperplasia (BPH) and an equal number of apparently healthy control subjects. The three groups were matched for age and town of residence in the greater Athens area. Steroid hormones, sex hormone-binding globulin, and insulin-like growth factor 1 (IGF-1) were measured in duplicate by radioimmunoassay in a specialized US centre. Statistical analyses were performed using multiple logistical regression. The results for IGF-1 in relation to prostate cancer and BPH were adjusted for demographic and anthropometric factors, as well as for the other measured hormones. There was no relation between IGF-1 and BPH, but increased values of this hormone were associated with increased risk of prostate cancer; an increment of 60 ng $ml^{-1}$ corresponded to an odds ratio of 1.91 with a 95% confidence interval of 1.00–3.73. There was also some evidence for an interaction between high levels of testosterone and IGF-1 in relation to prostate cancer. This finding suggests that, in addition to testosterone, IGF-1 may increase the risk of prostate cancer in humans.

Insulin-like growth factor 1 (IGF-1) is secreted mainly by the liver but is also produced in several other tissues in response to growth hormone (LeRoith et al, 1992). It has been documented that IGF-1 can act in an autocrine and paracrine manner to promote normal growth and malignant cellular proliferation (Daughaday, 1990; LeRoith et al, 1992). The importance of IGF-1 as a major growth-regulating molecule has been established for cells in culture (Goustin et al, 1986; LeRoith et al, 1992; Webster et al, 1996) and has also been suggested by studies in vivo (Ezzat and Melmed, 1991). IGF-1 and several of its binding proteins are produced by normal prostate cells (Cohen et al, 1991) as well as prostate cancer cells (Pietrzjowski et al, 1993; Kimura et al, 1996) and act locally through activation of IGF-1 receptors to stimulate cell proliferation (Angelloz-Nicoud and Binoux, 1995). In addition to its established autocrine and paracrine action, IGF-1 has important endocrine functions (LeRoith et al, 1992). However, the role, if any, of circulating IGF-1 in the aetiology of benign prostatic hyperplasia (BPH) and prostate cancer has not received sufficient attention.

We have previously reported results concerning the role of serum steroid hormones in the aetiology of BPH (Lagiou et al, 1997) and prostate cancer (Signorello et al, 1997) from a matched case–control study undertaken in Athens, Greece. Circulating IGF-1 was measured in the same sera, and we present the relevant results in this report.

*Received 22 January 1997*
*Revised 14 April 1997*
*Accepted 30 April 1997*

*Correspondence to:* D Trichopoulos, Department of Epidemiology, Harvard School of Public Health, 677 Huntington Avenue, Boston, Massachusetts 02115, USA

### SUBJECTS AND METHODS

In the context of a large, ongoing case–control study of diet in relation to prostate cancer and BPH, blood samples were collected during an 18-months period from 52 incident cases of histologically confirmed prostate cancer and 52 cases of BPH with matching for age (±1 year) and town of residence within the greater Athens area. Within the constraints imposed by this matching, cases of prostate cancer and BPH were randomly selected from among those enrolled in the larger study.

In order to choose appropriate controls, we identified day-care centres for the elderly that exist throughout the urban centres of Greece (KAPI). In these centres, healthy, elderly people meet for social interaction and entertainment. We approached attendees at KAPI centres in the same or neighbouring towns as those of the matched cancer/BPH pairs. One control was randomly selected for each matched cancer/BPH pair, again with matching for age within 1 year. Among the eligible controls, fewer than ten declined to participate and were replaced. In addition to blood, all study participants provided information on their exact age, height, weight and years of schooling.

Frozen serum samples were shipped from Athens to Beth Israel Hospital in Boston, USA, packed in dry ice. The coded samples arrived unthawed and in good condition and were analysed without knowledge of case–control status by laboratory personnel under the supervision of one of the investigators (CSM).

IGF-1 concentrations were measured, after ethanol extraction, by a commercially available radioimmunoassay kit (Nichols Institute, San Juan Capistrano, CA, USA). Testosterone (T), oestradiol (E2), and dehydroepiandrosterone sulphate (DHEAS) were measured by commercially available radioimmunoassay kits (Diagnostic Products, Los Angeles, CA, USA), and sex hormone-binding globulin (SHBG) concentrations were determined by a commercially available enzyme-linked immunosorbent assay (ELISA) (Wallac,

**Table 1** Distribution of 52 cases of incident prostate cancer, 52 cases of incident benign prostatic hyperplasia (BPH), and 52 healthy controls by age, years of schooling, and anthropometric variables

| Variable | Prostate cancer *n* (%) | BPH *n* (%) | Controls *n* (%) |
|---|---|---|---|
| Age (years) | | | |
| ≤69 | 20 (38.5) | 18 (34.6) | 21 (40.4) |
| 70–74 | 18 (34.6) | 19 (36.5) | 17 (32.7) |
| ≥75 | 14 (26.9) | 15 (28.9) | 14 (26.9) |
| Years of schooling | | | |
| 0–5 | 12 (23.1) | 11 (21.2) | 15 (28.9) |
| 6 | 15 (28.9) | 12 (23.1) | 20 (38.5) |
| 7–11 | 12 (23.1) | 11 (21.2) | 11 (21.2) |
| ≥12 | 13 (25.0) | 18 (34.6) | 6 (11.5) |
| Height (cm) | | | |
| <165 | 8 (15.4) | 4 (7.7) | 16 (30.8) |
| 165–169 | 12 (23.1) | 18 (34.6) | 13 (25.0) |
| 170–174 | 12 (23.1) | 14 (26.9) | 14 (26.9) |
| ≥175 | 20 (38.5) | 16 (30.8) | 9 (17.3) |
| Weight (kg) | | | |
| <70 | 13 (25.0) | 14 (26.9) | 13 (25.0) |
| 70–79 | 21 (40.4) | 21 (40.4) | 17 (32.7) |
| 80–89 | 12 (23.1) | 12 (23.1) | 16 (30.8) |
| ≥90 | 6 (11.5) | 5 (9.6) | 6 (11.5) |
| Body mass index (kg m$^{-2}$) | | | |
| <24 | 13 (25.0) | 17 (32.7) | 13 (25.0) |
| 24–26.99 | 20 (38.5) | 14 (26.9) | 11 (21.2) |
| 27–29.99 | 11 (21.2) | 18 (34.6) | 20 (38.5) |
| ≥30 | 8 (15.4) | 3 (5.8) | 8 (15.3) |

**Table 2** Spearman correlation coefficients between the measured hormones among healthy controls

| | IGF-1 | Testosterone | DHT | SHBG | DHEAS |
|---|---|---|---|---|---|
| IGF-1 | – | – | – | – | – |
| Testosterone | 0.10 | – | – | – | – |
| DHT | 0.03 | 0.34[a] | – | – | – |
| SHBG | –0.15 | 0.60[a] | 0.38[a] | – | – |
| DHEAS | 0.37[a] | 0.28[a] | 0.34[a] | –0.02 | – |
| Oestradiol | –0.01 | 0.31[a] | 0.27[a] | 0.11 | 0.38[a] |

[a]*P*-value <0.05; IGF-1, insulin-like growth factor 1; SHBG, sex hormone-binding globulin; DHT, dihydrotestosterone; DHEAS, dehydroepiandrosterone sulphate.

Gaithersburg, MD, USA). Dihydrotestosterone (DHT) concentrations were measured, after extraction, using commerically available radioimmunoassay kits (DSL International, TX, USA). All hormones were measured in duplicate, and the average of the two measurements for each hormone was used for data analyses. The sensitivities of the assays used were as follows: IGF-1, 13 ng ml$^{-1}$; T, 4.0 ng dl$^{-1}$; E2, 2.0 pg ml$^{-1}$; SHBG, 0.5 nmol l$^{-1}$; DHT, 4.0 pg ml$^{-1}$; DHEAS, 1.1 mg dl$^{-1}$. The intra-assay coefficients of variation were as follows: IGF-1, 2.4–3.0%; T, 4.0–7.0%; E2, 4.0–5.0%; SHBG, 1.4–1.8%; DHT, 3.1–6.2%; DHEAS, 6.0–9.8%. No significant cross-reactivity exists between the measured hormones. Cross-reactivity between IGF-1 and IGF-2 with the antiserum used in this assay has been shown to be 0.5%, whereas there is virtually no cross-reactivity between IGF-1 and other peptide hormones. IGF-1-binding proteins were removed through the extraction method before measuring IGF-1.

For the analyses, cases with prostate cancer and BPH cases were alternatively compared with the healthy controls. Statistical analyses were performed using stratification and modelling of the data by multiple logistic regression (Breslow and Day, 1980). Conditional and unconditional models were essentially identical. All *P*-values are two-tailed. The STATA statistical package (Stata Corporation, College Station, TX, USA) was used throughout.

### RESULTS

Table 1 presents descriptive demographical and anthropometrical measures of study participants in each of the three groups. These factors have been adjusted, using multiple logistic regression, in the evaluation of the hormonal correlates of prostate cancer and BPH. Table 2 shows Spearman correlation coefficients of the five hormones studied and SHBG in the controls. The associations between IGF-1, on the one hand, and the remaining five factors, on the other, are not strong, with the possible exception of that with DHEAS. Of particular interest is the lack of association between IGF-1 and DHT, because several of the BPH patients as well as some of the prostate cancer patients who had coexisting BPH could have taken in the past (despite exclusion protocol requirements) finasteride (a 5 α-reductase inhibitor that blocks the conversion of T to DHT).

© Cancer Research Campaign 1997

**Table 3** Mean value and standard error (SE) of steroid hormones and SHBG in the three study series

| Hormone | Prostate cancer | | BPH | | Controls | |
|---|---|---|---|---|---|---|
| | mean | (SE) | mean | (SE) | mean | (SE) |
| Testosterone (ng $dl^{-1}$) | 447.1 | (38.4) | 480.1 | (30.0) | 541.8 | (27.2) |
| Oestradiol (pg $ml^{-1}$) | 11.0 | (3.6) | 7.9 | (1.3) | 22.5 | (2.5) |
| DHT (pg $ml^{-1}$) | 161.8 | (20.5) | 180.6 | (21.3) | 634.9 | (59.5) |
| DHEAS (μg $dl^{-1}$) | 114.9 | (11.7) | 123.7 | (12.6) | 110.5 | (8.6) |
| SHBG (nmol $l^{-1}$) | 53.8 | (2.4) | 56.9 | (3.4) | 58.3 | (3.3) |

BPH, benign prostatic hyperplasia; DHT, dihydrotestosterone; DHEAS, dehydroepiandrosterone sulphate; SHBG, sex hormone-binding globulin.

**Table 4** Frequency distribution of incident cases of prostate cancer, benign prostatic hyperplasia (BPH) and healthy controls by marginal quartiles of IGF-1

| Variable | *n* | Mean | (SD) | Quartiles of IGF-1[a] 1 (*n*) | 2 (*n*) | 3 (*n*) | 4 (*n*) | *P*-value for linear trend[b] (unadjusted) |
|---|---|---|---|---|---|---|---|---|
| IGF-1 (ng $ml^{-1}$) | | | | | | | | |
| Prostate cancer | 51 | 160.3 | (68.2) | 8 | 13 | 13 | 17 | 0.01 |
| BPH | 50 | 146.0 | (68.2) | 13 | 10 | 16 | 11 | 0.13 |
| Controls | 52 | 124.7 | (58.6) | 17 | 18 | 8 | 9 | |

[a]Q1, ≤92.3; Q2, 92.4–135.0, Q3, 135.1–184.5; Q4, >184.5; [b]compared with controls. IGF-1, insulin-like growth factor 1.

**Table 5** Multiple logistic regression – derived adjusted odds ratios for prostate cancer and benign prostatic hyperplasia (BPH) by specified increment of IGF-1

| IGF-1 | $OR_{crude}$ | OR[a] | OR[b] | 95% CI[b] | *P*-value[b] |
|---|---|---|---|---|---|
| IGF-1 (per 60 ng $ml^{-1}$ increment) | | | | | |
| Prostate cancer vs control subjects | 1.71 | 1.52 | 1.91 | (1.00, 3.73) | 0.05 |
| BPH vs control subjects | 1.38 | 1.23 | 0.99 | (0.48, 2.06) | 0.99 |

[a]Adjusted for age, height, body mass index, and years of schooling; [b]adjusted for age, height, body mass index, years of schooling, SHBG and the four hormones listed in Table 3. IGF-1, insulin-like growth factor 1.

Table 3 presents a summary of the results that have been separately reported concerning levels of steroid hormones and SHBG in the three study series. In these data, after adjusting for age, height, body mass index, education and mutually among the hormones and SHBG, cases of prostate cancer were found to have higher levels of T ($P = 0.07$), lower levels of DHT ($P < 0.001$) and no remarkable differences in levels of E2 and SHBG compared with the healthy control subjects. Cases of BPH were observed to have higher levels of DHEAS ($P = 0.01$), but no significant difference in levels of SHBG, T, or E2 when compared with the same control subjects.

Tables 4 and 5 present the principal results of this study that focus on IGF-1. The crude analysis in Table 4 indicated that IGF-1 levels are substantially higher among patients with prostate cancer in comparison with controls, whereas among patients with BPH the elevation of IGF-1 is smaller and non-significant. After adjusting for demographical and anthropometrical risk factors as well as for the other hormones, the association between IGF-1 and prostate cancer is strengthened and remains statistically significant. In contrast, the weak association between IGF-1 and BPH disappears after adjustment for the same set of confounders (Table 5).

Among the steroid hormones that have been investigated in relation to prostate cancer, T stands out on the basis of quality-adjusted empirical evidence and biomedical credibility. Therefore, we evaluated whether T and IGF-1 may interact in relation to prostate cancer. We used median values of IGF-1 and T in the combined distribution of prostate cancer cases and controls to create four groups, using subjects with low values of both IGF-1 and T as the reference for categorical contrasts. The odds ratios (95% CI) were: 1.30 (0.19–8.86) for subjects with high T and low IGF-1; 2.97 (0.46–19.06) for subjects with low T and high IGF-1; 6.86 (0.75–62.56) for subjects with high T and high IGF-1. It appears that there may be an interaction of high levels of both IGF-1 and T in the causation of prostate cancer if the associations are indeed causal.

### DISCUSSION

There is circumstantial evidence that androgens play a role in the aetiology of prostate cancer and BPH. Androgens are essential for the growth and function of the prostate and can produce prostate cancer and BPH in experimental animals (Goustin et al, 1986; Webster et al, 1996). T is the dominant stimulus for prostatic

© Cancer Research Campaign 1997

growth, whereas adrenal androgens, including DHEAS and androstendione, are weaker androgens that can be converted to more potent ones (T and DHT) in several tissues including the prostate (Montie and Pienta, 1994; Geller, 1995). DHT, a hormone produced by reduction of T by 5 α-reductase, is the most potent intracellular androgen, but it is the intraprostatic rather than the circulating DHT that affects prostate growth (Geller, 1993; Montie and Pienta, 1994; Geller, 1995).

With respect to IGF-1, the evidence that implicates it in the aetiology of cancer of the prostate derives mostly from in vitro studies and pathophysiological considerations. Normal and malignant prostate cells produce IGF-1 and several of its binding proteins that can act in a paracrine or autocrine manner (Cohen et al, 1991; Pietrzjowski et al, 1993; Kimura et al, 1996). IGF-binding proteins are found abundantly in prostate secretions, and their serum levels have been reported to differ between patients with and without prostate cancer (Cohen et al, 1993; Kanety et al, 1993). Moreover, prostate cells express IGF-1 receptors and are very sensitive to stimulation by IGFs (Cohen et al, 1991; Kimura et al, 1996). In vitro activation of IGF-1 receptors induces proliferation of prostate cancer cells that is directly dependent on IGF availability and is modulated by IGF-binding proteins (Angelloz-Nicoud and Binoux, 1995). In addition, antisense RNA to IGF-1 receptor suppresses tumour growth and prevents invasion by rat prostate cancer cells in vivo (Burfeind et al, 1996). Finally, suramin, a drug that inhibits prostate cancer growth and is now being tested in clinical trials for prostate cancer, is thought to act, at least in part, by decreasing serum levels of IGF-1 and 2 (Miglietta et al, 1993).

There is scarce epidemiological literature concerning IGF-1 in relation to cancer in general and, to our knowledge, no epidemiological study has been published reporting on the relationship between IGF-1 and prostate cancer or BPH. Our findings suggest that IGF-1 may play a role in the aetiology of prostate cancer. This interpretation is strengthened by the lack of association between IGF-1 and BPH. Moreover, pathophysiological considerations and experimental evidence impart an element of biomedical credibility in the causal link between IGF-1 and prostate cancer. Suggestive evidence that IGF-1 may play a causal role in breast cancer (Weiderpass et al, 1997) can also be thought of as supportive for a similar link with respect to prostate cancer.

A straightforward causal interpretation is hindered by a number of considerations. Case–control investigations cannot satisfy the time sequence criterion for causality and cannot directly address the concern that the disease may alter levels of the hormones under investigation. Moreover, the present study is relatively small and statistical significance is not a guarantee that chance did not contribute to the generation of results. In addition, interrelations between serum hormone levels and recognized or unsuspected feedback mechanisms may contribute to residual confounding of unpredictable magnitude and direction. Finally, the strikingly reduced DHT levels in cases with prostate cancer and BPH, possibly a result of unreported 5 α-reductase inhibitor use by some patients, justifies concern, even although IGF-1 was unrelated to DHT in controls or, indeed, in subjects in any of the three study groups.

In conclusion, the results of the present study raise the possibility that IGF-1 may increase the risk of prostate cancer but provide no evidence that this hormone plays a role in the aetiology of BPH. The evidence of interaction between IGF-1 and T with respect to prostate cancer is statistically weak ($P = 0.09$), but it is biologically credible and offers additional opportunities for evaluating the hypotheses that emerge from these data.

### ACKNOWLEDGEMENTS

This study was supported in Greece by a grant from the Europe Against Cancer programme of the European Union, and in Boston by a grant to Harvard University from the Monsanto company. Lisa B Signorello is supported by a training award in cancer epidemiology from the US National Institutes of Health, National Cancer Institute (5T32CA09001-21). Christos S Mantzoros is supported by the Division of Endocrinology, Beth Israel Deaconess Medical Center and the Clinical Investigator Training Program of Harvard/MIT Health Sciences and Technology, in collaboration with Pfizer.

### REFERENCES

Angelloz-Nicoud P and Binoux M (1995) Autocrine regulation of cell proliferation by the insulin-like growth factor (IGF) and IGF binding protein-3 protease system in a human prostate carcinoma cell line (PC-3). *Endocrinology* **136**: 5485–5492

Breslow NE and Day NE (1980) *Statistical Methods in Cancer Research*. Vol. I *The Analysis of Case–Control Studies*, IARC Scientific Publication 32. International Agency for Research on Cancer: Lyon

Burfeind P, Chernicky CL, Rininsland F, Ilan J and Ilan J (1996) Antisense RNA to the type I insulin-like growth factor receptor suppresses tumor growth and prevents invasion by rat prostate cancer cells *in vivo*. *Proc Natl Acad Sci USA* **93**: 7263–7268

Cohen P, Peehl DM, Lamson G and Rosenfeld RG (1991) Insulin-like growth factors, IGF receptors and IGF binding proteins in primary culture of prostate epithelial cells. *J Clin Endocrinol Metabol* **73**: 401–407

Cohen P, Peehl DM, Stamey TA, Wilson KF, Clemmons DR and Rosenfeld RG (1993) Elevated levels of insulin-like growth factor binding protein-2 in the serum of prostate cancer patients. *J Clin Endocrinol Metab* **76**: 1031–1035

Daughaday WH (1990) The possible autocrine/paracrine and endocrine roles of insulin like growth factors of human tumors. *Endocrinology* **127**: 1–4

Ezzat S and Melmed S (1991) Are patients with acromegaly at increased risk for neoplasia? *J Clin Endocrinol Metab* **72**: 245–249

Geller J (1993) Basis for hormonal management of advanced prostate cancer. *Cancer* **71**: 1039–1045

Geller J (1995) Approach to chemoprevention of prostate cancer. *J Clin Endocrinol Metab* **80**: 717–719

Goustin AS, Leof EB, Shipley GD and Moses HL (1986) Growth factors and cancer. *Cancer Res* **46**: 1015–1029

Kanety H, Madjar Y, Dagan Y, Levi J, Papa MZ, Pariente C, Goldwaser R and Karasik A (1993) Serum insulin-like growth factor binding protein-2 (IGF BP-2) is increased and IGF BP-3 is decreased in patients with prostate cancer: Correlation with serum prostate specific antigen. *J Clin Endocrinol Metab* **77**: 229–233

Kimura G, Kasuya J, Giannini S, Honda Y, Mohan S, Kawachi M, Akimoto M and Fujita-Yamaguchi Y (1996) Insulin-like growth factor (IGF) system components in human prostatic cancer cell-lines: LNCaP, DU145, PC-3 cells. *Int J Urol* **3**: 39–46

Lagiou P, Mantzoros CS, Tzonou A, Signorello LB, Lipworth L and Trichopoulos D (1997) Serum steriods in relation to benign prostatic hyperplasia. *Oncology* (in press)

Leroith D, Clemmons D, Nissley P and Rechler MM (1992) NIH Conference. Insulin-like growth factors in health and disease. *Ann Intern Med* **116**: 854–862

Miglietta L, Barreca A, Repetto L, Constantini M, Rosso R and Boccardo F (1993) Suramin and serum insulin-like growth factor levels in metastatic cancer patients. *Anticancer Res* **13**: 2473–2476

Montie JE and Pienta K (1994) Review of the role of androgenic hormones in the epidemiology of benign prostatic hyperplasia and prostate cancer. *Urology* **43**: 892–898

Pietrzkowski Z, Mulholland G, Gomella L, Jameson BA, Wernicke D and Baserga R (1993) Inhibition of growth of prostatic cancer cell lines by peptide analogues of insulin-like growth factor 1. *Cancer Res* **53**: 1102–1106

Signorello LB, Tzonou A, Mantzoros CS, Lipworth L, Lagiou P, Hsieh C-c, Stampfer M and Trichopoulos D (1997) Serum steroids in relation to prostate cancer risk in a case–control study (Greece). *Cancer Causes Control* (in press)

Webster NJ, Resnik JL, Reichart DB, Strauss B, Haas M and Seely BL (1996) Repression of the insulin receptor promoter by the tumor suppressor gene product p53: a possible mechanism for receptor overexpression in breast cancer. *Cancer Res* **56**: 2781–2788

Weiderpass E, Gridley G, Persson I, Nyren O, Ekbom A, Hoover R and Adami HO (1997) Risk of endometrial and breast cancer in patients with diabetes mellitus. *Int J Cancer* **71**: 360–363

© Cancer Research Campaign 1997